# Storm Daniel Extreme Flood Event in Thessaly, Greece: Assessing the Pollution Status of the Impacted Coastal Marine Areas through Extended Screening of Emerging Contaminants Using LC-TIMS-HRMS

**DOI:** 10.1021/acs.estlett.5c00122

**Published:** 2025-03-04

**Authors:** Rallis Lougkovois, Georgios Gkotsis, Constantine Parinos, Ioannis Hatzianestis, Maria-Christina Nika, Alexandra Pavlidou, Nikolaos Thomaidis

**Affiliations:** † Hellenic Centre for Marine Research, Institute of Oceanography, 46.7 Km Athens-Sounio av., Mavro Lithari, 19013 Anavyssos, Attiki, Greece; ‡ National and Kapodistrian University of Athens, Department of Chemistry, Laboratory of Analytical Chemistry, University Campus, Zografou, 15771 Athens, Greece

**Keywords:** Pagasitikos Gulf, Aegean Sea, pharmaceutically active compounds, PFAS, industrial chemicals, plant protection products, climate change

## Abstract

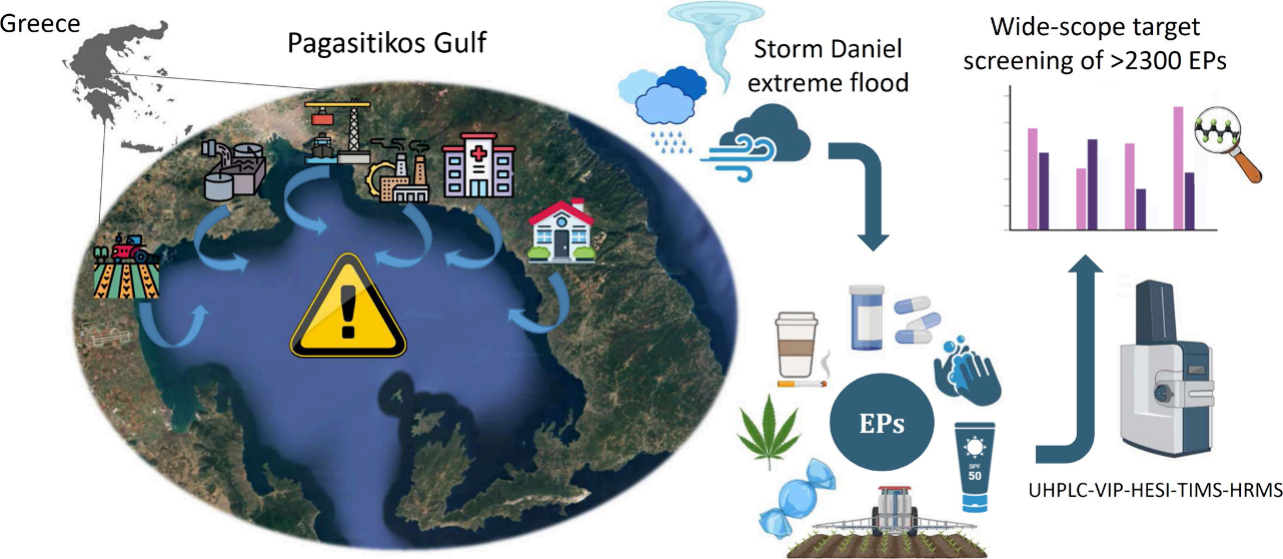

In this study, we investigate the pollution status in impacted seawaters and sediments of coastal areas in the region of Thessaly, central Greece, following the Storm Daniel extreme flood event in September 2023, a phenomenon classified as the deadliest Mediterranean tropical-like cyclone in recorded history and one of the costliest cyclones beyond the North Atlantic. For this, an ultra high-performance liquid-chromatography-based wide-scope target screening of more than 2300 LC-amenable emerging contaminants (ECs) was carried out utilizing the technique of TIMS-QTOF-MS. Our results highlight the fact that the extreme floodwater runoff resulted in an extensive transportation of terrestrial derived material from the impacted areas and a major transport of plant protection products, pharmaceuticals, including even illicit drugs, surfactants, industrial chemicals, and per- and polyfluorinated alkyl compounds (PFAS) at sea, as a result of the overflowing of local wastewater treatment plants and the destruction of agricultural, industrial, and port facilities. Overall, the phenomenon resulted in a major alteration of the classification of the determined ECs in seawaters and most importantly in sediments of the study area, with potential implications for their environmental quality status.

## Introduction

Extreme weather events, exacerbated by climate change, pose a severe threat for society, its economy, and the environment.^1−3^ The coastal marine ecosystem is among the most impacted by climate-driven extreme events, receiving numerous natural and anthropogenic stressors.^3−5^ Major flooding constitutes one of the most destructive consequences of extreme weather events. Besides their effects on the wider socioeconomic spectrum, floodwater run-offs can carry a wide range of organic contaminants at sea, inflicting potential hazardous implications for marine life.^6,7^ Among these, emerging contaminants (ECs) cover a wide range of chemical substances that reach the marine environment due to human-related activities. However, little information is available regarding their ecotoxicological impact and potential adverse effects. Recent advances in high-resolution mass spectrometric techniques allowed the study of the occurrence of different groups of ECs in the marine environment.^8−12^

**Figure 1:**
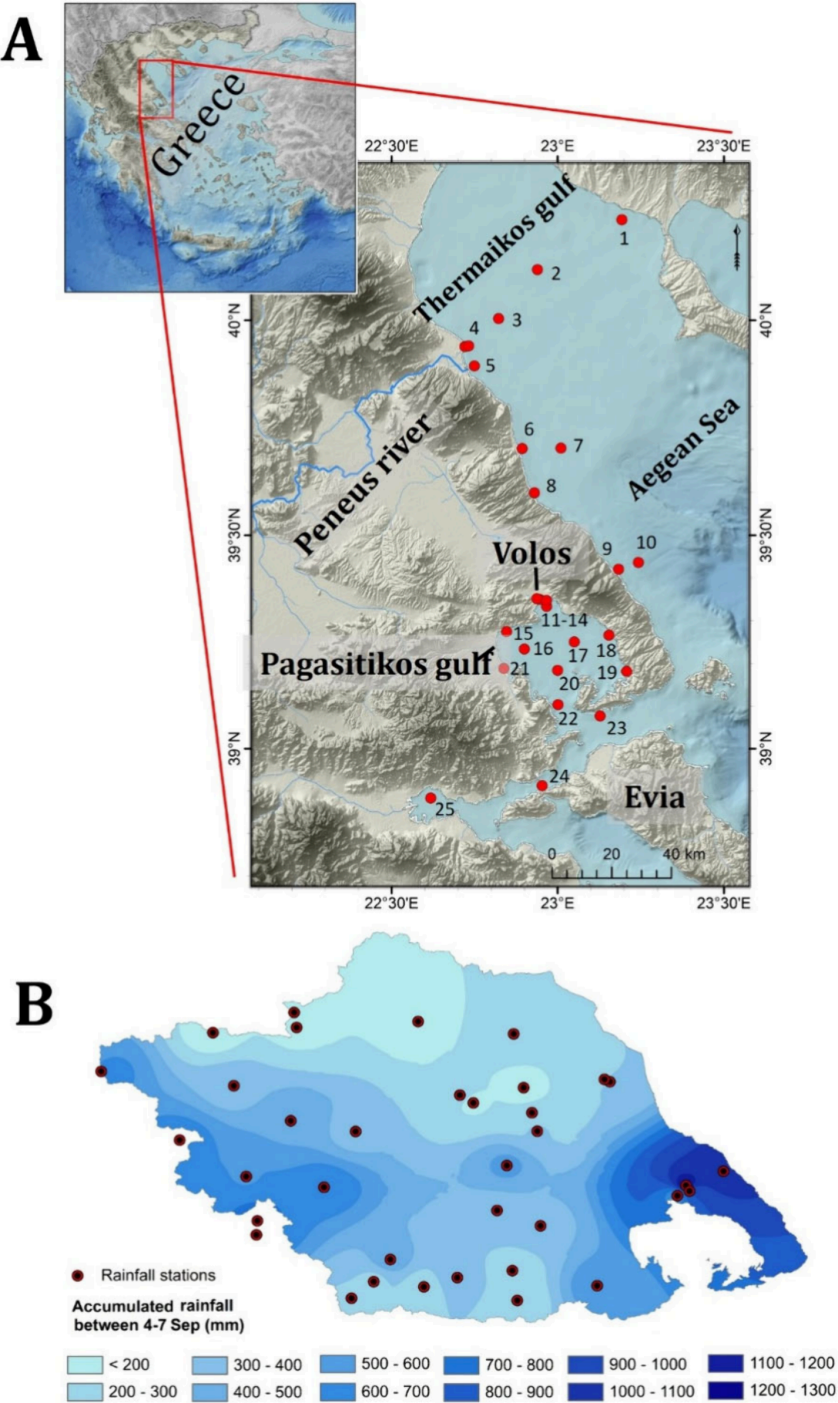
Location of sampling sites across the Pagasitikos Gulf and the NW Aegean Sea (A). Total accumulated rainfall from September 4th to 7th, 2023, during storm Daniel in the wider Thessaly region, central Greece (data adapted from Dimitriou et al.^14^) (B).

The Mediterranean region, a critical hotspot for climate-driven environmental change,^1^ faced an unprecedented drought-flood abrupt alternation over 2022–2023.^13^ On September 3–8th, 2023, storm Daniel caused extreme rainfall in the Thessaly region in central Greece (Figure 1a). The system then evolved into a Medicane (Mediterranean tropical-like cyclone), landing on the shores of Libya on September 10th, 2023, causing more than 11000 casualties in the city of Derna. Storm Daniel has been classified as the deadliest Medicane in recorded history and one of the costliest cyclones beyond the North Atlantic.^13^

Within Thessaly, rainfall exceeded 400 mm in most areas and 900 mm in the central/eastern regions, equal to their annual average values^14^ (Figure 1b). With a flooded area of 1150 km^2^,^15^ the phenomenon had devastating environmental and economic impact resulting in 18 casualties, destroying household/industrial/agricultural facilities, wastewater treatment plants (WWTPs), and ports, causing an extreme runoff at the Pagasitikos Gulf (Figure 1a). Moreover, rainfall reached an average of 360 mm over the Peneus River basin, resulting in a peak flow of 1950 m^3^/s at the river mouth and an outflow volume of around 1670 hm^3^ of water to the NW Aegean Sea^12^ (Figure 1a). Following storm Daniel, storm Elias also hit parts of the heavily flooded Thessaly on September 25–28th, 2023, with less force than storm Daniel since it landed southern on the island of Evia, although the capital city of Thessaly, Volos, flooded again, receiving 298 mm of rainfall.

Understanding the impact of extreme flood events on the marine environment is of critical importance on a global scale, related to the effective adaptation of management plans for the protection and restoration of the marine ecosystem under a changing climate.^1^ To the best of our knowledge, previous studies conducted in impacted coastal areas of the Mediterranean and worldwide following flash-flood events are limited, focusing on inorganic parameters,^16^ plastics/microplactics,^17−19^ trace metals,^20,21^ and legacy organic contaminants (PAHs),^22^ while only Castaño-Ortiz et al. (2023)^7^ reported on the occurrence of a limited number of pharmaceuticals in Mar Menor lagoon following a flash-flood event in 2019.

In this study, a comprehensive ultra high-performance liquid-chromatography-based wide-scope target screening of more than 2300 LC-amenable compounds and their transformation products (TPs) was carried out, utilizing the technique of trapped ion mass spectrometry-hybrid quadrupole time-of-flight-mass spectrometry (TIMS-QTOF-MS), to assess the chemical burden in seawater and sediments of the impacted coastal marine areas following the Thessaly extreme flash-flood events, also incorporating a pre- and postflooding comparison.

## Methods and Materials

### Study Area

Pagasitikos is a semi enclosed gulf in central Greece with a mean depth of 69 m. It covers ∼520 km^2^ with a catchment area of ∼1458 km^2^. It is characterized by limited freshwater inflow occurring mainly north–northwest in Volos and Almyros during winter and spring^23,24^ (Figure 1a). The Pagasitikos Gulf is an ecosystem under anthropogenic pressure. The only major city of Volos (∼140000 inhabitants) along with its well-developed industrial zone and port is located to the north. The industrial zone includes metal processing plants, food production/packaging facilities, wood processing units, and a cement plant. The Volos WWTP applies chemical and biological treatment to industrial and domestic wastes. The gulf also receives various anthropogenic inputs related to intense agricultural/farming activities, mainly in its western part.^23^

### Sampling and Analysis

Following the extreme flood events, a sampling campaign was conducted in the impacted coastal marine areas during October 4–8th, 2023. Seawater samples were collected from various depths at a grid of 25 stations along with corresponding sediments (Figure 1a; Table S.3). All seawater samples were collected with 12 L Niskin bottles mounted on a rosette system. In all cases, 2.5 L of seawater were immediately transferred to clean amber-glass bottles with Teflon-lined caps. Samples were acidified using HCl 2M, transferred to the laboratory, and stored at 4°C pending analysis. As for marine sediments, all samples (undisturbed top-1 cm) were collected using a stainless-steel Box Corer with a surface area of 40 × 40 cm, wrapped in prefurnaced aluminum foil and stored at −20 °C pending analysis.

For the pretreatment of all collected samples previously reported methodologies were followed.^25,26^ Chromatographic analysis was carried out utilizing ultra high-potential liquid chromatography (Elute LC series, Bruker Daltonics), coupled to a hybrid TIMS-QTOF mass spectrometer (timsTOF Pro 2, Bruker Daltonics). Wide-scope target screening was performed by conducting two separate runs for positive and negative vacuum insulated probe-heated electrospray ionization (VIP-HESI) modes. The targeted compound set includes more than 2300 LC-amenable ECs, such as 1127 pharmaceutically active compounds (PhACs) and their TPs, 84 per- and polyfluorinated alkyl substances (PFAS), 57 other industrial chemicals, and 702 plant protection products (PPPs) and their TPs.

Acquired data were treated by applying strict identification criteria for the targeted compounds. QA/QC procedures were followed to ensure the quality assurance of received data. In brief, procedural blank samples were treated for both environmental matrices by using the same sample preparation protocol. Moreover, the evaluation of possible cross contamination during sampling was conducted by using Milli-Q water as a field blank. Seawater samples were preconcentrated via solid phase extraction (SPE), while sediments were treated via ultrasonic-assisted extraction (UAE). The HRMS analysis was operated in both positive and negative ionization modes with Data Independent Acquisition, with the aim to extend the chemical domain accessible to wide-scope target screening. Detailed information on the methodology and the complete data set of the targeted compounds is provided in the Supporting Information.

## Results and Discussion

**Figure 2:**
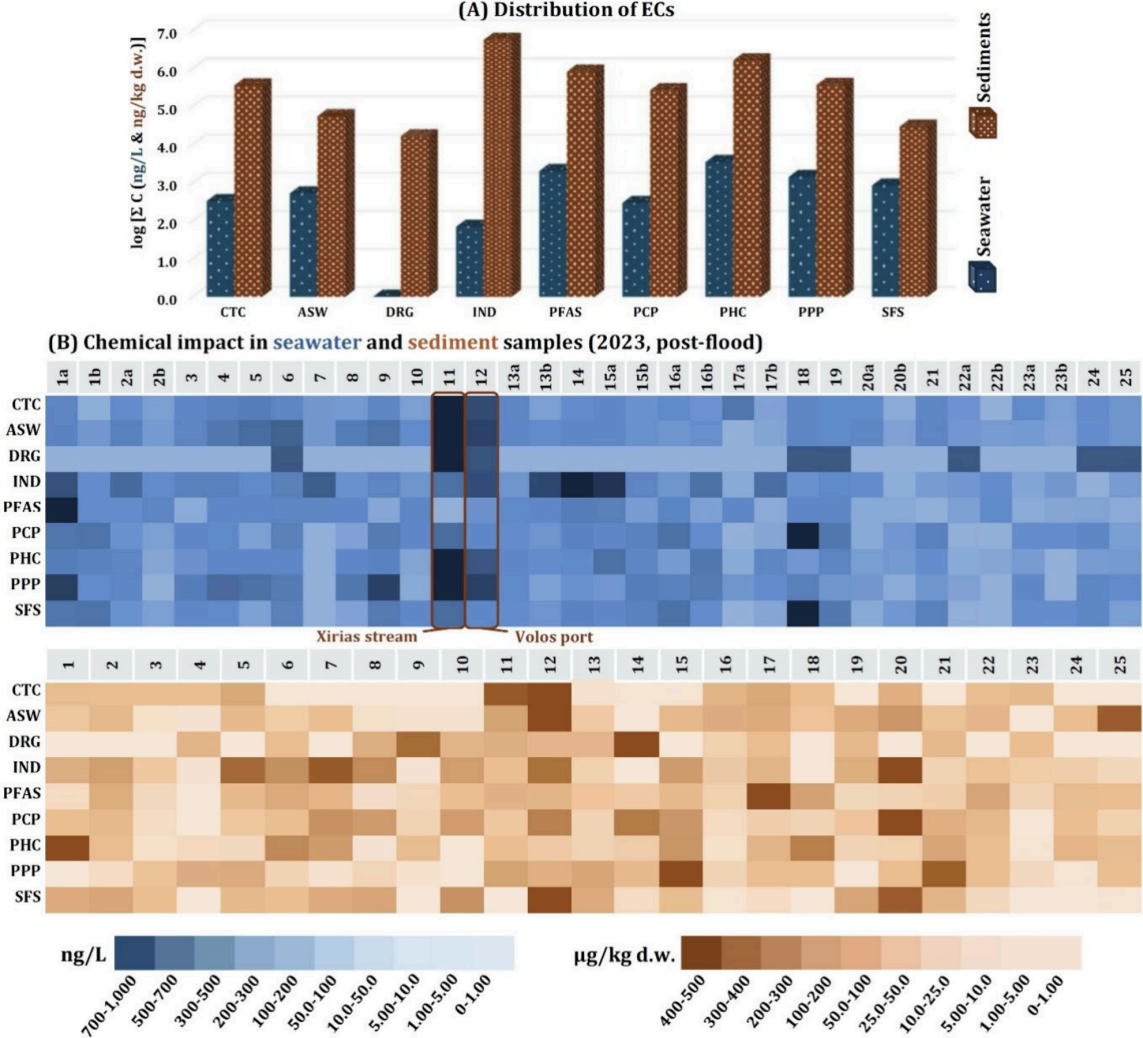
Logarithmic values of total EC concentrations per chemical class in postflood seawaters and sediments (A) and heatmaps for seawater and sediment sampling locations encumbrance as cumulative concentrations of ECs postflood (B). Locations with 2 seawater samples marked as “a” and “b” yielded two samples, one close to the surface (a) and one close to the sea bottom (b). Abbreviations as are follows: CTC, coffee- and tobacco-related compounds; ASW, artificial sweeteners; DRG, illicit drugs and drugs of abuse; IND, industrial chemicals; PFAS, per- and polyfluorinated alkyl compounds; PCP, personal care products; PHC, pharmaceuticals and TPs; PPP, plant protection products and TPs; SFS, surfactants.

Wide-scope target analysis revealed the presence of 282 ECs in the postflood samples. 232 analytes were detected in seawater and 92 in sediments, 42 of which were commonly detected in both matrices. Compounds detected solely in seawater, such as PhACs and PPPs were generally more polar (log *K*_ow_ < 1) than those detected in sediments, mainly consisting of industrial chemicals, semipolar pharmaceuticals, and long carbon-chain surfactants (Figure 2a).

Stations 11 (Xirias stream) and 12 (Volos port) were the most chemically burdened, in terms of cumulative concentration levels of ECs, with 115 compounds detected in each case and cumulative concentrations of ECs reaching up to 1.15 and 0.715 μg/L, respectively. These samples contained a variety of coffee- and tobacco-related compounds and pharmaceuticals (Figure 2). Concerning sediments, Stations 12 (Volos port) and 15 (Anhialos) were the most chemically burdened sites. 38 compounds were detected in Volos port, with a total concentration of ECs reaching up to 634 ng/kg. These substances were mainly comprised of coffee- and tobacco-related compounds, summing up to 113 ng/kg. 33 analytes were determined in Anhialos, reaching up to 978 ng/kg, comprised mainly by semipolar industrial chemicals, such as PFAS and plasticizers, summing up to 277 ng/kg (Figure 2).

**Figure 3:**
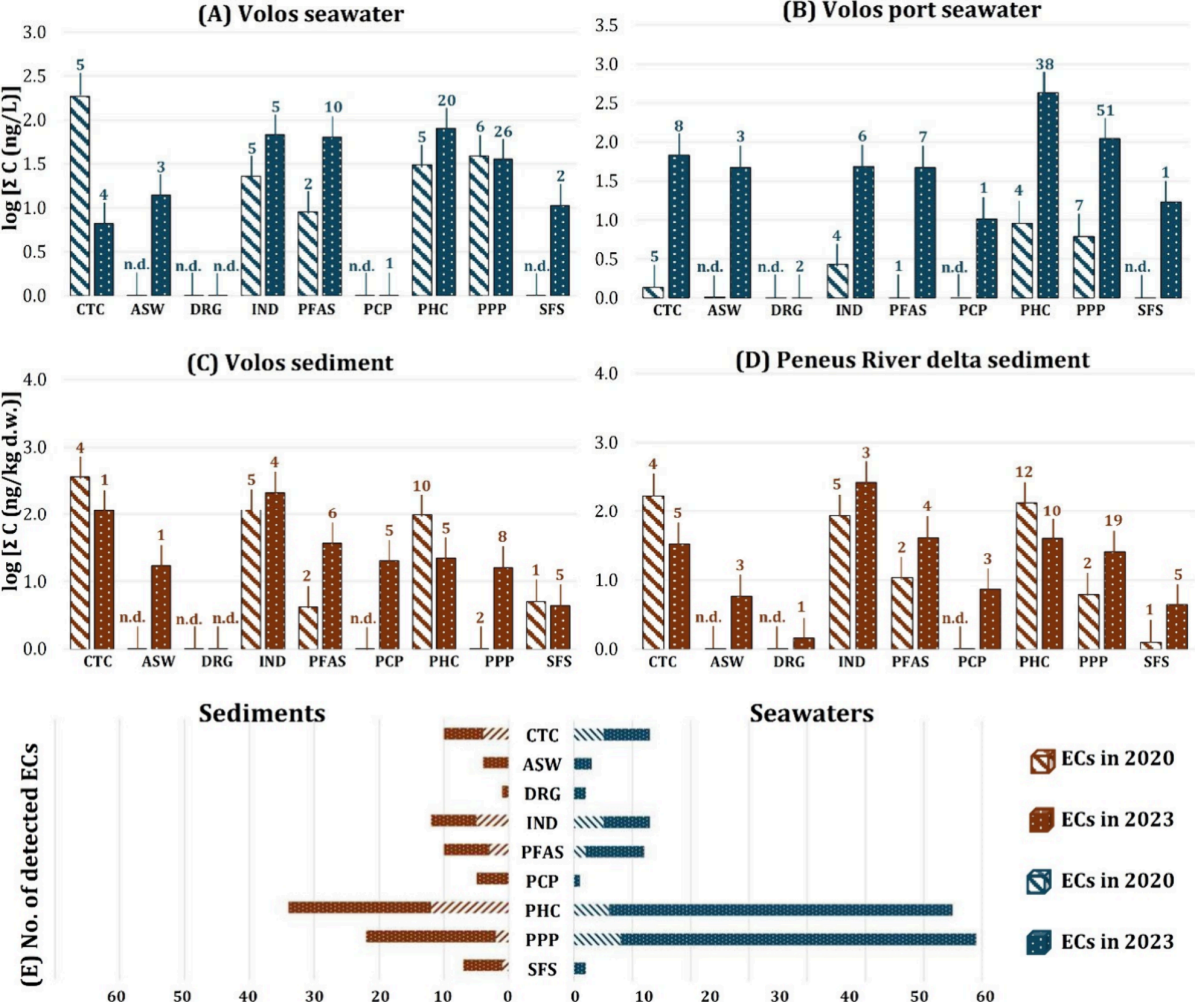
Comparison of the chemical imprint between 2020 and 2023 for the common seawater sampling locations integrating the number of the determined ECs (A,B). Comparison of the chemical imprint between 2020 and 2023 for the common sediment sampling locations, integrating the number of the determined ECs (C,D). Total number of ECs per chemical class in seawater and sediment samples for both 2020 and 2023 sampling campaigns (E). Abbreviations are as follows: CTC, coffee- and tobacco-related compounds; ASW, artificial sweeteners; DRG, illicit drugs and drugs of abuse; IND, industrial chemicals, PFAS, per- and polyfluorinated alkyl compounds; PCP, personal care products; PHC, pharmaceuticals and TPs; PPP, plant protection products and TPs; SFS, surfactants.

Although the marine environmental status of the Pagasitikos Gulf has been widely studied over past decades,^23^ previous data on ECs are not available in the literature, while published data on the Greek Seas are limited, focusing on pharmaceuticals in seawater.^27,28^ To compare the pre- and postflood event chemical burden in the impacted coastal areas, unpublished data concerning the same set of targeted compounds from a limited number of seawater and sediment samples collected in 2020 at Volos, Volos Port and Peneus River delta were considered herein (Figure 3a–d; Table S.3, S.6, and S.7). Samples from 2020 were treated with the same sample preparation procedures and analyzed with the same instrumentation and parameters as the postflood samples.

Concerning postflood seawater samples, 172 PhACs were detected in the study area. In Stations 12 (Volos port) and 13 (Volos), 53 PhACs were detected, whereas only 6 compounds were detected in 2020 (Figure 3a,b). Among these, the antihypertensive substance losartan, in concentrations between 2.69 and 11.6 ng/L, with a 100% frequency of detection (FoD), was not detected in 2020. Losartan reportedly causes adverse effects regarding cell life cycle and acceleration of immune system failure of *P. perna* mussels,^29^ which are widely cultivated in Greece and indigenously inhabit river estuaries in the Mediterranean.

Interestingly, the illicit drug cocaine and its human metabolite benzoylecgonine were determined postflood in the most chemically burdened samples of Xirias stream and Volos port. Literature suggests that a ratio of cocaine/benzoylecgonine concentrations not exceeding 0.75 is linked to human consumption of said narcotics. Herein, a ratio value of 0.35 ties the findings both to cocaine consumption and raw wastewater reaching the sea likely due to WWTPs overflowing at the Volos port area.^30,31^

Furthermore, in Volos port, two psychoactive compounds were detected in 2020, namely venlafaxine, along with its human consumption metabolite o-desmethyl-venlafaxine, in concentrations ranging between 0.343 and 4.25 ng/L. Postflood, these two compounds were also determined in comparable concentrations, along with 20 additional psychoactive substances, with total concentrations reaching up to 116 ng/L (Figure 3b). Among them, 4 benzodiazepines, bromazepam, citalopram, flurazepam, and the latter’s metabolite desalkyl-flurazepam (100% FoD), were detected, summing up to 4.78 ng/L.

A total of 18 PFAS, out of the 84 included in our database, were detected in tested seawaters. Legacy PFAS like perfluorooctanesulfonic acid (PFOS) and next-generation ones like 4,8-dioxa-3*H*-perfluorononanoic acid (ADONA) were omnipresent in postflood samples (100% FoD). In 2020 at the Volos Port-Volos, only two PFAS were determined, namely, perfluoroheptanoic acid (PFHpA) and perfluorohexanoic acid (PFHxA), in concentrations not exceeding 1.43 and 0.698 ng/L, respectively. In postflood seawaters of the same stations, an increase was evident in both previously determined PFAS concentrations to a maximum of 7.46 ng/L for PFHpA and 1.90 ng/L for PFHxA. The presence of 16 additional PFAS was confirmed in 2023 (Figure 3a,e).

Finally, 84 PPPs were detected postflood in seawater samples of the study area. At the Volos Port-Volos area (Stations 12 and 13; Figure 1) during 2020, 8 PPPs were determined in seawater, with a total concentration reaching up to 54.1 ng/L (Figure 3a,b). Among them atrazine (33% FoD in 2020, as opposed to 100% FoD postflood), along with one of its TPs, atrazine-2-hydroxy (100% FoD during both sampling campaigns). Postflood, these substances were determined in similar levels, equaling 47.4 ng/L, with an additional 53 PPPs at a total concentration of 498 ng/L. These chemicals mainly consisted of herbicides (23 compounds) and fungicides (15 compounds) including alachlor, diuron, prometryn (caparol), and simazine (Figure 3e).

In the studied sediments, 28 PhACs were detected, which tend to differ in polarity from those present in seawater, like quinolone antibiotics cinoxacin and orbifloxacin, uniquely detected in sediments, which have estimated log *K*_ow_ values of 1.59 and 2.37, respectively, attributed to greater affinity with the nonpolar sediment layer (Figure 3e). Their detection in concentrations up to 11.9 ng/kg is possibly linked to sorption mechanisms onto microplastics, which then precipitate, as previously reported.^32^

During the postflood sampling, a new psychoactive substance (NPS) was detected, 5-methoxy-*N*,*N*-dimethyltryptamine (5-MeO-DMT), which is classified as a psychedelic of the tryptamine subclass, in concentrations ranging between 1.06 and 7.51 ng/kg (16% FoD). NPSs of said subclass have been previously reported in Greek WWTP influents^33^ and their postflood occurrence is likely due to large volumes of untreated wastewater reaching the sea.

Postflood sediments contained a total of 15 PFAS, with total concentrations ranging between 4.06 and 239 ng/kg (Figure 2a). Sediments from stations 17, 18, and 22 (Figure 1) were the most burdened regarding PFAS, with total concentrations summing up to 239, 77.3, and 95.4 ng/kg, respectively. The 6:2-fluorotelomer sulfonic acid (6:2-FTS) and perfluorooctanesulfonamide (PFOSA) were also determined, at concentrations reaching up to 26.4 and 0.560 ng/kg, respectively.

Finally, during the 2020 sampling, two PPPs were determined in sediments of the Volos and Peneus River delta areas (Figure 1; Figure 3c–e), triclopyr and *N*,*N*-diethyl-m-toluamide (DEET) both with 100% FoD in concentrations ranging between 0.417 and 5.73 ng/kg. Only DEET was detected in 2023 with 50% FoD. However, 18 compounds were exclusively determined postflood, in concentrations ranging between 16.1 and 25.3 ng/kg. Among them was prometryn (caparol), also determined in seawaters, at concentrations ranging between 0.084 and 0.171 ng/kg (11% FoD).

The chemical quality of European coastal waters is regulated by the Water Framework Directive (WFD).^34,35^ Seawater samples collected postflood contained three priority substances, namely alachlor (33% FoD), diuron (100% FoD), and imidacloprid (33% FoD), with their maximum concentrations being 0.250, 0.825, and 1.55 ng/L, respectively. Although concentration levels for alachlor and diuron are 2–3 orders of magnitude lower than the Annual Average (AA) environmental quality standards (EQS) levels, established by Directive 2013/39/EU, imidacloprid’s concentration surpasses the AA (i.e., 0.680 ng/L). These compounds have been restricted for more than a decade, so their presence in the studied seawaters could be due to their persistent nature and continuous accumulation in the terrestrial ecosystem. As for PFAS, their total concentrations, expressed as PFOA-equivalents,^35^ ranged between 0.331 and 107 ng/L, with 65% of the tested samples exceeding the AA EU EQS value (i.e., 4.40 ng/L). Concerning sediments, no EQS values for the studied ECs are considered by the EU WFD.

### Implications for the Environmental Quality Status of the Impacted Marine Areas

Concluding, the wide-scope target screening employed herein provided an extended insight toward the extraneous chemical impact in seawater and sediments of the study area. However, further research is needed to assess the determined EC mobility, toxicity, and possible bioaccumulation. As the inclusion of priority pollutants in legislation worldwide is a dynamic process, HRMS methodologies provide the ability to analyze samples retrospectively. Thus, the results reported in this study could be considered as a reference for future scientific research in this area and beyond.

## Supporting Information Available

Detailed information about the seawater and sediment sample preparation for chromatographic analysis, instrumental analysis, QA/QC procedures, data treatment, wide scope target screening database, gradient elution program for chromatographic analysis, internal standards used, location of the sampling sites across the Pagasitikos Gulf and the NW Aegean Sea, results from HRMS analysis on seawater and sediment samples collected in 2023, and results from HRMS analysis on seawater and sediment samples collected in 2020 (PDF)
