# Supplementary material for: Storm Daniel Extreme Flood Event in Thessaly, Greece: Assessing the Pollution Status of the Impacted Coastal Marine Areas through Extended Screening of Emerging Contaminants Using LC-TIMS-HRMS
Source: Environ Sci Technol Lett. 2025 Mar 4;12(4):432–9. doi: 10.1021/acs.estlett.5c00122 (PMC11984090; doi:10.1021/acs.estlett.5c00122)
Supplement: Supplementary file 1 — ez5c00122_si_001.pdf [file ez5c00122_si_001.pdf]

Storm Daniel extreme flood event in Thessaly, Greece: Assessing the pollution status of the impacted coastal marine areas through extended screening of emerging contaminants using LC-TIMS-HRMS

Rallis Lougkovois[1,2], Georgios Gkotsis[2], Constantine Parinos[1,*], Ioannis Hatzianestis[1], Maria-Christina Nika[2], Alexandra Pavlidou[1], Nikolaos Thomaidis[2,**]

[1] Hellenic Centre for Marine Research, Institute of Oceanography, 46.7 Km Athens-Sounio av., Mavro Lithari, 19013, Anavyssos, Attiki, Greece

[2] National and Kapodistrian University of Athens, Department of Chemistry, Laboratory of Analytical Chemistry, University Campus, Zografou, 15771, Athens, Greece

Supplementary Information

Corresponding authors:

* Hellenic Centre for Marine Research, Institute of Oceanography – Constantine Parinos, email: ksparinos@hcmr.gr, tel: +30 2291076372

** National and Kapodistrian University of Athens, Department of Chemistry, Laboratory of Analytical Chemistry – Nikolaos Thomaidis, email: ntho@chem.uoa.gr, tel: +30 2107274317

Sample preparation for chromatographic analysis

To identify the chemical imprint in the tested seawaters, previously reported generic sample preparation protocol with slight modifications was applied for seawater samples[1], while for sediments, a previously in-house developed method was followed[2]. Both methodologies aimed to extract as many semi-polar to polar, thermally unstable, LC-amenable organic micropollutants, along with their respective TPs and metabolites, as possible. Key points with detailed information regarding sample preparations are described subsequently:

Seawater samples preparation for LC-TIMS-QTOF-MS wide-scope target screening

1L of each sample was stored in the dark at 4°C until extraction. Sample pH was adjusted to 6.5 (±0.2) with a few drops of HCl 0.1M and ammonium hydroxide 25% v/v. An isotopically labelled standards (I.S.) mix was spiked in each sample. A respective spiking procedure with the standards mixture took place, producing 6 spiked samples. Samples were left to stand for 15 min and then sample clean-up and pre-concentration was realized by SPE. Layered mixed-mode cartridges consisting of Oasis HLB (200mg) and a mixture of Strata-X-AW (weak anion exchanger), Strata-X-CW (weak cation exchanger) and Isolute ENV+ (350mg of total mixture) are used. Conditioning of the cartridges was performed with **3mL methanol** followed by **3mL water**. Elution of the analytes from the sorbent materials was performed using a **basic solution** [6mL of ethyl acetate/methanol (50/50 v/v) containing 2% v/v ammonium hydroxide], followed by an **acidic solution** [(4mL of ethyl acetate/methanol (50/50 v/v) containing 1.7% v/v formic acid]. The extract was then evaporated to dryness under a gentle nitrogen stream (at 40°C) and reconstituted to a final volume of **200μL** (methanol/ Milli-Q, 50/50 v/v). The extract was filtered directly into a 2 mL glass vial using a syringe fitted with a 0.22μm RC membrane filter and was stored in the freezer (-20°C) pending analysis.

Cartridge assembly:

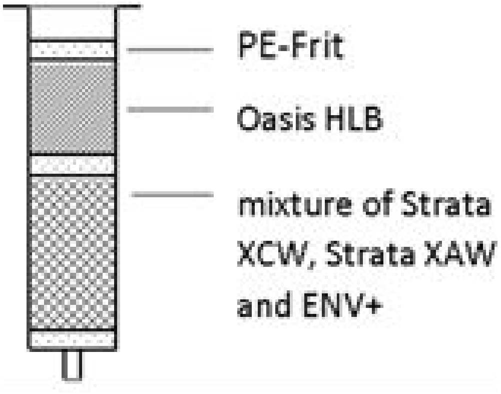

Sediment samples preparation method for LC-TIMS-QTOF-MS wide scope screening

1g of lyophilized sample was stored in a 15mL centrifuge tube in the dark at room temperature until extraction. An I.S. mix solution was spiked in each sample and a respective spiking procedure with the standards mixture takes place, producing 6 spiked samples. Samples were left to stand for 15min.

Extraction step:

3mL of extraction solvent mixture was added to the sample via Pasteur pipette. Samples were shaken by hand for 1 min, followed by a visual check that the entire sediment mass is wet and then shaken by vortex for another 1min. Ultrasonic Assisted Extraction (UAE) at **50°C** for **15min** (UAE's water bath temperature needs to be adjusted to 50oC prior to the extraction) took place and then samples were centrifuged at **4,000rpm** for **10min**. Supernatant liquid was collected in 15 mL glass test tubes. The extraction steps were repeated **2 more times** (total of **9mL** supernatant liquid collection). The extracts were then evaporated to dryness under a gentle nitrogen stream (at 40°C) and reconstituted to a final volume of **200µL** (methanol/ Milli-Q, 50/50 v/v). The extracts were filtered directly into 2mL glass vials using syringes fitted with 0.22µm RC membrane filters and were stored in the freezer (-20°C), pending analysis.

Extraction solvent preparation:

Methanol/Milli-Q 50/50 v/v (0.5% v/v formic acid and 0.1% w/v EDTA), prepared the day of the extraction and stored in the dark until use

Instrumental analysis

Extracted analytes were separated using ultra-high potential liquid chromatography (UHPLC) (Elute LC series, Bruker Daltonics, Bremen, Germany), coupled to a hybrid trapped ion mobility-quadrupole time-of-flight (TIMS-QTOF) spectrometer (timsTOF Pro 2, Bruker Daltonics, Bremen, Germany). The gradient elution conditions are presented in **Table S1**:

Table S.1. Gradient elution program parameters

Time (min)	Flow (mL/min)	%A	%B
0.00	0.200	96.0	4.0
0.10	0.200	96.0	4.0
1.00	0.200	81.7	18.3
2.50	0.223	50.0	50.0
14.00	0.400	0.1	99.9
16.00	0.480	0.1	99.9
16.10	0.480	96.0	4.0
19.00	0.480	96.0	4.0
19.10	0.200	96.0	4.0
20.00	0.200	96.0	4.0

Solvents used for mobile phases in **positive (+)** ionization mode were:

A. H_2O/MeOH 99/1 v/v, 0.01% formic acid, 0.005M ammonium formate

B. MeOH, 0.01% formic acid, 0.005M ammonium formate

Solvents used for mobile phases in **negative (–)** ionization mode were:

A. H_2O/MeOH 99/1 v/v, 0.005M ammonium acetate

B. MeOH, 0.005M ammonium acetate

Prior to instrumental analysis, calibration of both mobility and mass accuracy took place. Regarding mobility calibration, CCS values of certain compound ions included in an IMS calibration mix were evaluated and accepted if %ΔCCS value did not exceed 1% (>99% accuracy). As far as mass accuracy is concerned, masses of cluster sodium formate ions produced in the ion source were considered. Up to par mass accuracy was accepted if Δm/z values did not exceed ±0.1mDa (>99% accuracy).

Wide-scope target screening was performed by conducting two separate reversed-phase chromatographic runs for positive and negative vacuum insulated probe - heated electrospray ionization (VIP-HESI) modes. Auto sampler injection volume was set at 1μL for positive ionization and at 3μL for negative ionization mode, ensuring up to par peak intensity for identified analytes and their respective fragment ions. A Data Independent Acquisition (DIA) mode, called broadband Collision Induced Dissociation (bbCID) was applied. In DIA, low collision energy (4eV) produces a full scan spectrum, followed by high collision energy (25eV) producing MS/MS fragment ion spectra. DIA data were used for quantification purposes in the wide-scope target screening of organic micropollutants and their TPs.

Samples were analyzed with TIMS-ON modes, making use of the instrumentation's added value, providing a 4th dimension of separation, granting the ability to effectively separate isomeric compounds without the need to apply specific chromatographic parameters[4]. This instrumentation also amplifies peak capacity and increases sensitivity. Data received from the TIMS-ON analysis mode are translated into cross-collision section values (CCS)[3,4]. Experimental CCS values are then considered for %ΔCCS calculation, adding an

additional identification parameter to the already strict ones provided by the HRMS application.

QA/QC procedures

In the interest of ensuring quality assurance of received data, QA/QC procedures were followed. Procedural and field blanks were analyzed along with each batch of samples. A sample for each batch, which was expected to be the least chemically burdened was used for spiking, producing 6 levels of spiked samples, used for quantification purposes, aiming to bypass different matrices effects. Quantification was conducted based on the spike curve.

Analytes were confirmed for determination only if their relative area was equal or above 150% of each blank sample's relative area, provided that the analyte is also confirmed in the blanks. Every sample and blank analyzed was also spiked with a mixture of deuterated internal standards to evaluate the instrumentation's sensitivity during analysis and facilitate analyte quantifications. The list of chosen internal standards utilized for this work, based on their efficient representation of analytes included in our dataset is presented in **Table S.2**:

Table S.2. List of Internal Standards utilized in RPLC for quantification purposes

I.S.	Formula	m/z (Da)	RT (min)	CCS (Å^2)
Amitriptyline D3	$C_{20}D_3H_{20}N$	281.2092	7.40	168.45
Atenolol D7	$C_{14}D_7H_{15}N_2O_3$	274.2143	3.49	159.39
Atrazine D5	$C_8D_5H_9ClN_5$	221.1324	7.59	150.30
Bisphenol A D16	$C_{15}D_{14}H_2O_2$	241.1956	7.57	161.84
Caffeine D9	$C_8D_9HN_4O_2$	204.1441	4.32	141.18
Cetirizine D8	$C_{21}D_8H_{17}ClN_2S$	397.2129	8.16	200.17
Citalopram D4	$C_{20}D_4H_{17}FN_2O$	329.1962	5.72	181.99

I.S.	Formula	m/z (Da)	RT (min)	CCS (Å^2)
Diazepam D5	$C_{16}D_5H_8ClN_2O$	290.1103	8.94	166.70
Diuron D6	$C_9D_6H_4Cl_2N_2O$	239.0620	7.82	151.55
Flunixin D3	$C_{14}D_3H_8F_3N_2O_2$	300.1034	8.90	164.24
Mefenamic acid D3	$C_{15}D_3H_{12}NO_2$	245.1364	11.22	155.82
Meloxicam D3	$C_{14}D_3H_{10}N_3O_4S_2$	355.0609	6.74	178.94
Metoprolol D7	$C_{15}D_7H_{18}NO_3$	275.2347	4.66	173.40
Sertraline D3	$C_{17}D_3H_{14}Cl_2N$	309.0999	8.05	169.32
Sulfadiazine D4	$C_{10}D_4H_6N_4O_2S$	255.0848	3.83	155.15
Sulfamerazine D4	$C_{11}D_4H_8N_4O_2S$	269.1005	4.12	160.06
Tramadol D6	$C_{16}D_6H_{19}NO_2$	270.2335	4.64	162.57
Venlafaxine D6	$C_{17}D_6H_{21}NO_2$	284.2491	5.54	172.27

A QC sample was also analyzed for every 6 samples, ensuring proper performance of instrumentation, along with a pure mobile phase solvents mixture to assess possible carry-over effects. At the start of each sequence, a System Suitability Test (SST) sample was also processed, containing 20 analyte standards, representative of the analytes included in the database, regarding retention time, exact mass and CCS values. Use of said SST sample is deemed mandatory to evaluate instrumental stability and proper functionality during analysis.

Data treatment

Acquired raw data were treated using the TASQ software (Bruker Daltonics). The occurrence of more than 2,300 analytes from different chemical classes (i.e. pharmaceutical compounds, industrial chemicals, plant protection products, surfactants etc.) was investigated using HRMS, by applying strict identification criteria. Possession of reference standard aliquots for each determined analyte provides the ability to conduct a wide-scope target

screening of compounds from different chemical groups. These parameters include mass accuracy (±2mDa), retention time shift (±0.2min), isotopic pattern [mSigma score < 100 (mSigma is a fit score provided by the software, regarding the experimental spectrum versus the theoretical one, considering the mass accuracy and relevant intensity of isotopes. The smaller the mSigma value the better. A score not exceeding 100 is considered acceptable)], qualifier ions detection (every single mandatory ion included in the database for each compound determined should be present and have the same chromatographic peak shape as the principal ion. Mandatory ions are the ones produced by MS and MS/MS scan modes, whose peak intensity exceeds 50% of the precursor ion's intensity) and %ΔCCS values matching (±2%), ensuring further minimization of false positive selection. Each analyte determined was also verified by analyzing reference standards, as deemed necessary for wide-scope target screening analysis.

Wide scope target screening database

More than 2,300 analytes are included in the HRMS target screening database of the Laboratory of Analytical Chemistry, Department of Chemistry, National and Kapodistrian University of Athens, Greece, such as 1,127 pharmaceuticals and their TPs, 84 per- and polyfluorinated alkyl substances (PFAS), 57 other industrial chemicals, 702 plant protection products and their TPs. Compound identifiers included are either already available in online databases, such as PubChem or Chemspider, while parameters still not available in literature are included in the database as experimental values, derived from direct infusions of reference standard aliquots into the MS/MS system.

Analytes from a variety of chemical classes are included in the database, such as pharmaceuticals, personal care products, drugs of abuse, coffee and tobacco related compounds, industrial chemicals, plant protection products, surfactants, artificial sweeteners

and naturally occurring compounds. Among them are priority pollutants included in European legislation for the preservation of the marine environment, while the majority of analytes are classified as emerging contaminants, not yet prioritized or regulated by national and supranational Law.

Complete analyte information is included in the National and Kapodistrian University of Athens' database by Thomaidis et al. (2024)[5].

Table S.3. Location of the sampling sites across the Pagasitikos Gulf and the NW Aegean Sea

No.	Station name	Latitude	Longitude	Station depth (m)	Sampling depth (m)	Sediment sampling
1	Nea Moudania	40° 13.955	23° 11.610	49	a. 2<br>b. 49	√
2	Central Thermaikos gulf	40° 07.020	22° 56.220	78	a. 2<br>b. 78	√
3	Peneus River delta out	40° 00.224	22° 49.377	76	a. 2<br>b. 76	√
4	Peneus River delta 1	39° 56.428	22° 43.940	31	2	√
5	Peneus River delta 2	39° 53.634	22° 44.972	47	2	√
6	Agiokampos	39° 42.125	23° 00.529	96	2	√
7	Agiokampos out	39° 42.146	22° 53.483	56	2	√
8	Pelion	39° 35.919	22° 55.833	33	2	√
9	Agios Ioannis	39° 25.041	23° 10.480	47	2	√
10	Agios Ioannis out	39° 25.166	23° 14.582	269	2	√
11	Xirias stream out	39° 21.103	22° 56.239	8	2	√
12	Volos port	39° 20.995	22° 56.700	11	2	√
13	Volos	39° 20.016	22° 57.997	33	a. 2<br>b. 32	√
14	Anavros	39° 20.794	22° 57.921	20	2	√
15	Anhialos	39° 16.455	22° 50.814	28	a. 2<br>b. 28	√
16	Pagasitikos gulf west	39° 13.994	22° 53.988	58	a. 2<br>b. 58	√

No.	Station name	Latitude	Longitude	Station depth (m)	Sampling depth (m)	Sediment sampling
17	Pagasitikos gulf east	39º 15.002	22º 02.956	96	a. 2<br>b. 96	√
18	Afissos	39º 15.972	22º 09.251	74	2	√
19	Milina	39º 10.888	22º 12.426	61	2	√
20	Pagasitikos gulf central	39º 11.059	22º 59.950	81	a. 2<br>b. 81	√
21	Almiros	39º 11.382	22º 50.482	10	2	√
22	Trikeri	39º 06.223	23º 00.145	81	a. 2<br>b. 81	√
23	South Pelion	39º 04.584	23º 07.716	77	a. 2<br>b. 77	√
24	Diavlos	38º 54.777	22º 57.165	56	2	√
25	Maliakos Gulf	38º 52.988	22º 37.148	18	2	√

Detected compounds

Detected compounds per chemical class and sub-class, along with their minimum, median, average and maximum values are presented in the following tables (**Tables S.4** and **S.5**) for seawater and sediment samples. Analyte frequence of detection percentage and limits of detection and quantification are also reported for both environmental matrices.

Table S.4. Determined analytes in post-flood **seawater** samples (N=35) collected in 2023. **Abbreviations:** BQL: Below Quantification Limit, FoD: Frequence of Detection

Analyte Name	Classification	Sub class	Minimum (ng/L)	Median (ng/L)	Average (ng/L)	Maximum (ng/L)	% FoD	LOD (ng/L)	LOQ (ng/L)
Anabasine	Coffee & Tobacco Related Compounds	Stimulants	BQL	2.89	4.04	15.0	80%	0.666	2.00
Caffeine			BQL	3.15	9.93	65.5	51%	0.230	0.689
Cotinine			0.743	5.11	7.79	20.2	11%	0.186	0.559
Hydroxy-Cotinine			BQL	2.41	3.03	12.2	49%	0.248	0.743
Harman			BQL	1.10	1.36	2.39	23%	0.216	0.649
Nicotine			BQL	0.736	1.44	4.84	23%	0.0413	0.124
Theobromine			BQL	9.79	9.79	14.4	11%	0.202	0.605
Theophylline			BQL	5.39	5.39	6.15	11%	0.793	2.38
Acesulfame	Food Additives	Artificial Sweeteners	1.48	4.01	5.22	19.0	94%	0.105	0.316
Cyclamic acid			1.36	1.36	1.43	1.58	9%	0.284	0.852
Saccharin			2.75	7.14	8.77	33.7	97%	0.160	0.479
Sucralose			BQL	3.85	4.57	10.5	43%	1.00	3.01
Benzoylecgonine	Illicit Drugs	Ecgonines	BQL	BQL	BQL	0.391	6%	0.113	0.338
Cocaine			BQL	BQL	BQL	BQL	6%	0.0653	0.196
2-Amino-Benzothiazole	Industrial Chemicals & TPs	Corrosion Inhibitors	BQL	0.114	0.120	0.165	23%	0.0917	0.275
3(2H)-one-1-2-Benzothiazole			BQL	0.192	0.189	0.247	51%	0.0578	0.173
1-H-Benzotriazole			BQL	2.87	3.48	7.07	40%	0.685	2.05
5.6-Desmethyl-Benzotriazole			BQL	1.04	1.23	2.39	37%	0.302	0.906
Tolytriazole			BQL	0.925	0.946	1.19	80%	0.223	0.670

Analyte Name	Classification	Sub class	Minimum (ng/L)	Median (ng/L)	Average (ng/L)	Maximum (ng/L)	% FoD	LOD (ng/L)	LOQ (ng/L)
Perfluorononanoic Acid-4,8-Dioxa-3H (ADONA)			0.270	0.278	0.278	0.286	6%	0.0433	0.130
2-Perfluorohexyl Ethanoic Acid (FHEA 6:2)			BQL	BQL	BQL	BQL	9%	0.0601	0.180
6:2 Fluorotelomer Sulfonic Acid (FTS 6:2)			BQL	4,55	106	815	51%	0.709	2.13
Perfluorobutanoic Acid (PFBA)			16.7	39.2	36.7	63.4	34%	0.884	2.65
Perfluoroheptanoic Acid (PFHpA)			BQL	7.65	9.87	34.1	69%	0.485	1.46
Perfluorohexanoic Acid (PFHxA)			BQL	4.64	8.48	37.8	57%	0.233	0.698
Perfluorohexanesulfonic Acid (PFHxS)		PFASs	0.156	0.801	0.844	1.62	20%	0.0461	0.138
Perfluorononanoic Acid (PFNA)			BQL	1.52	2.62	8.10	60%	0.319	0.957
Perfluorooctanoic Acid (PFOA)			BQL	2.88	3.35	17.2	100%	0.164	0.491
Perfluorooctanesulfonic Acid (PFOS)			0.116	0.371	0.528	3.12	83%	0.0570	0.171
Perfluorooctane Sulfonamide (PFOSA)			0.237	0.690	1.75	6.14	23%	0.0666	0.200
Perfluoropentanoic Acid (PFPeA)			BQL	14.0	20.5	33.4	26%	0.137	0.411
Perfluoropentane Sulfonic Acid (PFPeS)			BQL	BQL	BQL	BQL	20%	0.190	0.569
Desmethylphthalate		Plasticizers	BQL	0.276	0.850	2.18	17%	0.0632	0.190
Lauramidopropylbetaine	Personal Care Products	Skin Cleansers	BQL	10.1	15.6	49.1	80%	0.0364	0.109
4-AAA (4-Acetamido-Antipyrine)	Pharmaceuticals &	Analgesics &	BQL	0.644	0.597	0.661	29%	0.151	0.454

Analyte Name	Classification	Sub class	Minimum (ng/L)	Median (ng/L)	Average (ng/L)	Maximum (ng/L)	% FoD	LOD (ng/L)	LOQ (ng/L)
4-FAA (4-Formyl-Amino-Antipyrine)	TPs	Anaesthetics	BQL	BQL	BQL	BQL	23%	4.47	13.4
Acetamiprid			BQL	0.670	1.72	11.7	66%	0.0636	0.191
Cinchocaine			BQL	0.167	0.479	2.47	31%	0.0344	0.103
Embutramide			BQL	BQL	BQL	BQL	3%	0.118	0.355
Lidocaine			BQL	0.923	0.923	0.962	9%	0.192	0.577
Paracetamol			BQL	33.8	47.4	114	23%	2.15	6.45
Phenacetin			BQL	0.705	0.705	0.705	6%	0.469	1.41
Phenazone			BQL	0.347	0.354	0.535	34%	0.0957	0.287
Prilocaine			BQL	1.86	2.00	2.68	34%	0.292	0.875
Tetracaine			BQL	0.569	0.594	0.807	11%	0.114	0.341
Tramadol			BQL	0.127	0.127	0.141	11%	0.0362	0.109
Di-nor-Tramadol			0.815	0.815	0.815	0.815	3%	0.194	0.582
o-Desmethyl-Tramadol			BQL	2.60	2.84	5.70	29%	0.309	0.928
Albendazole		Anthelmintics	BQL	BQL	BQL	BQL	3%	0.475	1.42
Albendazole Sulfone			BQL	BQL	BQL	BQL	3%	0.0493	0.148
Fenbendazole			BQL	1.77	1.77	4.50	37%	0.186	0.558
2-Amino-Flubendazole			BQL	0.952	0.952	1.47	11%	0.0894	0.268
Mebendazole-Amine			BQL	BQL	BQL	BQL	11%	0.276	0.827
Ponazuril			0.878	2.47	2.47	4.07	6%	0.146	0.439
5-Hydroxy-Thiabendazole			BQL	BQL	BQL	BQL	3%	0.167	0.502
Triclabendazole			BQL	9.36	9.36	9.36	6%	0.299	0.896

Analyte Name	Classification	Sub class	Minimum (ng/L)	Median (ng/L)	Average (ng/L)	Maximum (ng/L)	% FoD	LOD (ng/L)	LOQ (ng/L)
Deacetyldiltiazem		Antiarrhythmics	BQL	BQL	BQL	BQL	3%	0.390	1.17
Disopyramide			BQL	BQL	BQL	BQL	3%	0.0417	0.125
Propafenone			BQL	0.330	0.730	2.65	51%	0.0500	0.150
Sotalol			1.27	1.99	1.99	2.71	6%	0.847	2.54
Azithromycin		Antibiotics	BQL	1.46	3.44	10.2	20%	0.133	0.399
Baquiloprim			15.3	15.3	15.3	15.3	3%	0.980	2.94
Erythromycin A			BQL	BQL	BQL	BQL	3%	0.437	1.31
Norfloxacin			BQL	0.960	0.960	0.960	9%	0.109	0.328
Flumequine			BQL	BQL	BQL	BQL	11%	0.107	0.320
Isoniazide			BQL	BQL	BQL	BQL	6%	0.251	0.752
Nigericin			BQL	BQL	BQL	BQL	3%	1.47	4.40
Oxolinic Acid			BQL	1.01	1.02	1.23	11%	0.125	0.375
Robenidine			BQL	BQL	BQL	BQL	3%	0.146	0.438
Salinomycin			BQL	BQL	BQL	BQL	3%	1.38	4.15
Sulfadiazine			BQL	0.868	0.868	0.868	11%	0.201	0.604
Sulfisozole			BQL	0.213	0.373	0.888	14%	0.0471	0.141
Tilmicosin			2.16	3.64	6.67	14.2	9%	0.717	2.15
Trimethoprim			BQL	BQL	BQL	BQL	6%	0,118	0,354
Metformin		Antidiabetic Agents	BQL	40.6	65.4	566	100%	3.90	11.7
Pioglitazone			BQL	0.528	0.651	0.906	17%	0.0830	0.249
Vildagliptin			BQL	2.79	2.79	3.28	11%	0.471	1.41
Carbamazepine		Antiepileptics	BQL	0.423	0.531	1.82	100%	0.0723	0.217

Analyte Name	Classification	Sub class	Minimum (ng/L)	Median (ng/L)	Average (ng/L)	Maximum (ng/L)	% FoD	LOD (ng/L)	LOQ (ng/L)
10.11-Epoxide-Carbamazepine			BQL	0.153	0.177	0.269	26%	0.0423	0.127
10-Hydroxy-Carbamazepine			BQL	BQL	BQL	BQL	6%	0.0628	0.188
Lamotrigine			BQL	BQL	BQL	BQL	3%	0.122	0.365
Levetiracetam			BQL	1.75	1.86	2.56	40%	0.364	1.09
Pregabalin			BQL	17.8	16.3	29.1	20%	0.310	0.931
Griseofulvin		Antifungals	BQL	5.23	5.23	5.87	11%	0.467	1.40
Isoconazole			BQL	5.54	5.54	5.54	11%	0.353	1.06
Miconazole			BQL	5.40	5.40	5.40	11%	0.350	1.05
Brompheniramine		Antihistamine Agents	BQL	1.14	1.10	2.66	20%	0.0804	0.241
Cetirizine			BQL	BQL	BQL	BQL	11%	0.160	0.480
Chlorpheniramine			BQL	BQL	BQL	BQL	9%	1.34	4.03
Etofylline			BQL	5.60	5.60	6.54	11%	1.47	4.42
Ketotifen			BQL	BQL	BQL	BQL	3%	0.222	0.665
Loratadine			BQL	1.73	1.73	2.82	11%	0.146	0.437
Promethazine			2.36	5.80	16.6	107	26%	0.717	2.15
Xylometazoline			BQL	BQL	BQL	BQL	3%	0.0739	0.222
Acebutolol		Antihypertensives	BQL	0.131	0.125	0.180	14%	0.0391	0.117
Atenolol			0.970	1.22	1.22	1.47	6%	0.294	0.882
Flecainide			BQL	0.200	0.200	0.252	9%	0.0401	0.120
Irbesartan			0.649	1.61	5.57	19.1	17%	0.144	0.433
Losartan			0.418	2.69	4.17	15.0	31%	0.127	0.380
Metoprolol			3.43	5.75	5.75	8.07	6%	0.0758	0.227

Analyte Name	Classification	Sub class	Minimum (ng/L)	Median (ng/L)	Average (ng/L)	Maximum (ng/L)	% FoD	LOD (ng/L)	LOQ (ng/L)
Minoxidil			BQL	2.20	5.99	29.2	51%	0.269	0.806
Pindolol			BQL	1.50	1.50	1.90	23%	0.359	1.08
Sitagliptin			BQL	4.64	5.31	7.02	14%	1.24	3.71
Triamterene			BQL	BQL	BQL	BQL	6%	0.334	1.02
Valsartan			BQL	8.77	15.8	34.8	37%	1.59	4.77
Clopidogrel		Antiplatelet Agents	BQL	BQL	BQL	BQL	11%	0.324	0.971
Lopinavir		Antiretrovirals	1.17	1.65	1.65	2.13	6%	0.150	0.450
Cimetidine		Antiulcers	BQL	2.66	2.70	3.91	40%	0.502	1.50
Metoclopramide			BQL	BQL	BQL	BQL	3%	0.287	0.861
Bromazepam		Benzodiazepines	BQL	BQL	BQL	BQL	3%	0.448	1.34
Desalkyl-Flurazepam			BQL	BQL	BQL	BQL	3%	0.357	1.07
Bunitrolol		Beta Blockers	BQL	2.15	2.15	2.44	11%	0.502	1.51
Carazolol			BQL	0.243	0.243	0.305	9%	0.0593	0.178
Propranolol			BQL	BQL	BQL	BQL	23%	0.297	0.891
Ipratropium		Bronchodilating Agents	BQL	1.92	1.92	1.92	6%	1.28	3.84
Salmeterol			BQL	BQL	BQL	BQL	3%	0.209	0.627
Memantine		Dementia Treatment Agents	BQL	0.434	0.434	0.581	11%	0.0664	0.199
Rivastigmine			BQL	BQL	BQL	BQL	20%	0.530	1.59
Hydrochlorothiazide		Diuretics	3.08	3.69	3.69	4.30	6%	0.137	0.410
Diclofenac		NSAIDs	BQL	BQL	BQL	BQL	3%	0.594	1.78
Mefenamic Acid			BQL	BQL	BQL	BQL	3%	5.17	15.5

Analyte Name	Classification	Sub class	Minimum (ng/L)	Median (ng/L)	Average (ng/L)	Maximum (ng/L)	% FoD	LOD (ng/L)	LOQ (ng/L)
Nabumetone			BQL	BQL	BQL	BQL	3%	0.770	2.31
Niflumic Acid			BQL	0.779	0.779	1.13	57%	0.285	0.856
Amisulpiride			BQL	0.742	0.770	1.32	11%	0.0719	0.216
Amitriptyline			BQL	1.29	1.55	3.26	49%	0.249	0.747
Amitriptyline-Oxide			BQL	BQL	BQL	BQL	6%	0.185	0.556
Chlorpromazine			BQL	11.2	11.2	11.2	6%	0.983	2.95
Citalopram			BQL	0.667	0.707	0.919	20%	0.0785	0.236
Norcitalopram			BQL	0.634	0.661	0.795	17%	0.102	0.305
Clomipramine			BQL	8.84	8.84	8.84	9%	0.590	1.77
Doxepine			BQL	2.03	2.03	2.03	9%	0.362	1.09
Droperidol			BQL	5.21	5.21	7.08	9%	1.09	3.26
Fluoxetine		Psychoactive Substances	1.36	1.55	2.16	3.58	9%	0.239	0.716
Haloperidol			BQL	1.95	2.61	5.92	23%	0.187	0.562
Maprotiline			BQL	1.49	1.87	2.88	11%	0.412	1.24
Paroxetine			BQL	2.71	2.71	2.71	9%	0.369	1.11
Quetiapine			BQL	BQL	BQL	BQL	3%	0.0597	0.179
Sertraline			BQL	8.30	8.30	8.30	9%	0.585	1.75
Sulpiride			BQL	BQL	BQL	0.206	23%	0.0599	0.180
Trazodone			BQL	1.04	1.12	2.10	17%	0.201	0.604
Venlafaxine			BQL	BQL	BQL	BQL	20%	0.228	0.685
o-Desmethyl-Venlafaxine			BQL	2.33	2.33	2.78	11%	0.191	0.572
Zimelidine			BQL	BQL	BQL	BQL	9%	0.899	2.70

Analyte Name	Classification	Sub class	Minimum (ng/L)	Median (ng/L)	Average (ng/L)	Maximum (ng/L)	% FoD	LOD (ng/L)	LOQ (ng/L)
Hordenine		Stimulants	BQL	BQL	BQL	3.59	23%	0.884	2.65
Mefexamide			BQL	BQL	BQL	BQL	3%	0.141	0.424
Dinitolmide		Veterinary Drugs	BQL	BQL	BQL	BQL	3%	0.0700	0.210
Florfenicol			BQL	0.550	0.550	0.554	29%	0.161	0.482
Lufenuron			1.09	1.09	1.09	1.09	3%	0.0910	0.273
Monensin			3.69	3.69	3.69	3.69	3%	1.05	3.15
Nequinate			BQL	1.63	1.63	2.71	9%	0.159	0.478
Azoxystrobin	Plant Protection Products & TPs	Fungicides	BQL	1.23	1.77	6.97	100%	0.0945	0.283
Azoxystrobin Acid			BQL	0.375	0.374	0.826	29%	0.0428	0.128
Boscalid			BQL	0.627	1.43	6.22	40%	0.0917	0.275
BTS 44596 (Prochloraz metabolite)			0.261	1.41	2.79	14.6	51%	0.0622	0.187
Carbendazim			BQL	0.162	0.208	0.352	43%	0.0379	0.114
CGA 321113 (Trifloxystrobin metabolite)			BQL	BQL	BQL	0.584	9%	0.159	0.478
4-Hydroxy-Chlorothalonil			BQL	BQL	BQL	BQL	9%	0.187	0.562
Dimethomorph			BQL	BQL	BQL	BQL	11%	0.188	0.563
Dimoxystrobin			BQL	BQL	BQL	BQL	3%	0.107	0.321
Dodine			0.199	0.199	0.199	0.199	3%	0.0349	0.105
Epoxiconazole			BQL	0.198	0.198	0.198	9%	0.0623	0.187
Fenbuconazole			BQL	BQL	BQL	BQL	17%	0.199	0.596
Fludioxonil			BQL	BQL	BQL	BQL	40%	0.126	0.379
Fluopyram			BQL	0.201	0.406	1.81	51%	0.0475	0.143

Analyte Name	Classification	Sub class	Minimum (ng/L)	Median (ng/L)	Average (ng/L)	Maximum (ng/L)	% FoD	LOD (ng/L)	LOQ (ng/L)
Fluxapyroxad			BQL	1.27	1.27	1.37	37%	0.177	0.532
Imazalil			BQL	0.673	0.673	0.873	23%	0.0821	0.246
Ipconazole			BQL	BQL	BQL	BQL	3%	0.172	0.517
Metalaxyl			BQL	0.553	0.752	1.50	49%	0.114	0.343
Metconazole			BQL	BQL	BQL	BQL	3%	0.203	0.608
Octhilinone			BQL	BQL	BQL	BQL	11%	0.295	0.884
Penconazole			0.739	0.739	0.739	0.739	3%	0.205	0.616
Prochloraz			BQL	1.20	1.20	1.20	6%	0.125	0.375
Spiroxamine			BQL	4.08	3.81	8.75	17%	0.127	0.382
Tebuconazole			BQL	3.54	3.14	4.51	29%	0.377	1.13
Tetraconazole			BQL	0.407	0.407	0.663	17%	0.100	0.301
Thiabendazole			BQL	0.234	0.226	0.324	17%	0.0333	0.100
Triflumizole			0.799	0.799	0.799	0.799	3%	0.0807	0.242
Carbanilide		Growth Regulators	BQL	BQL	BQL	BQL	14%	0.0705	0.212
5-Nitro-Guaiacol			0.275	0.698	0.665	0.989	11%	0.0777	0.233
Alachlor		Herbicides	BQL	BQL	BQL	BQL	3%	0.166	0.499
Ametryn			BQL	35.8	35.8	35.8	37%	7.48	22.4
Atrazine			BQL	0.583	0.647	1.76	100%	0.108	0.323
2-Hydroxy-Atrazine			0.162	0.337	0.328	0.694	69%	0.0451	0.135
Desethyl-Atrazine			BQL	BQL	BQL	BQL	11%	0.486	1.46
Desisopropyl-Atrazine			BQL	1.85	1.99	4.69	89%	0.369	1.11
Bentazone			BQL	0.516	0.668	1.42	57%	0.0447	0.134

Analyte Name	Classification	Sub class	Minimum (ng/L)	Median (ng/L)	Average (ng/L)	Maximum (ng/L)	% FoD	LOD (ng/L)	LOQ (ng/L)
CGA 142110 (Fluazifop-p-Butyl metabolite)			0.286	0.404	0.459	0.744	11%	0.0868	0.260
CGA 177960 (Diafenthiuron metabolite)			0.285	0.938	0.938	1.59	6%	0.0731	0.219
Chloridazone			BQL	BQL	BQL	BQL	14%	0.200	0.600
Desphenyl-Methyl-Chloridazone			BQL	BQL	BQL	BQL	3%	0.0694	0.208
Chlorotoluron			BQL	0.235	0.235	0.235	11%	0.157	0.470
Dimethenamid			BQL	BQL	BQL	BQL	9%	0.0615	0.185
Dinoterb			BQL	0.135	0.193	0.309	57%	0.0900	0.270
Diuron			BQL	0.164	0.283	0.825	49%	0.0471	0.141
Fluometuron			BQL	2.06	5.07	41.0	77%	0.0410	0.124
Linuron			BQL	1.46	1.46	1.59	11%	0.111	0.334
Metolachlor			BQL	0.673	2.08	12.4	83%	0.0364	0.109
Metolachlor-ESA			BQL	0.725	0.697	0.991	29%	0.113	0.340
Nicosulfuron			BQL	1.13	2.16	4.92	20%	0.151	0.454
Pretilachlor			BQL	0.424	0.424	0.424	11%	0.0790	0.237
Prometryn (Caparol)			BQL	0.351	0.559	2.82	97%	0.0487	0.146
Propazine			BQL	0.835	0.796	0.925	43%	0.162	0.485
Quinclorac			BQL	BQL	BQL	BQL	14%	0.163	0.490
Sebuthylazine			BQL	1.04	1.09	2.09	20%	0.0442	0.133
Simazine			BQL	9.76	10.5	27.5	100%	1.80	5.41
Simetryn			0.121	0.202	0.225	0.486	20%	0.0335	0.101
Terbuthylazine			BQL	0.563	0.795	3.37	54%	0.0586	0.176

Analyte Name	Classification	Sub class	Minimum (ng/L)	Median (ng/L)	Average (ng/L)	Maximum (ng/L)	% FoD	LOD (ng/L)	LOQ (ng/L)
Desethyl-Terbuthylazine			BQL	7.33	8.10	19.1	100%	1.37	4.11
Chlorantraniliprole		Insecticides	BQL	4.25	5.20	10.4	20%	0.315	0.944
Clothianidin			BQL	0.238	0.238	0.238	6%	0.0549	0.165
Coumaphos			0.567	0.745	0.745	0.923	6%	0.111	0.333
Diflubenzuron			0.601	0.601	0.601	0.601	3%	0.0541	0.162
Disulfoton-Sulfoxide			BQL	7.67	7.67	7.67	6%	1.50	4.50
Fenthion-Sulfoxide			BQL	BQL	BQL	BQL	26%	0.0367	0.110
Fipronil			BQL	0.304	0.304	0.304	34%	0.203	0.608
Fipronil-Sulfone			BQL	0.264	0.264	0.264	6%	0.176	0.528
Icaridin			BQL	1.52	1.52	2.06	29%	0.653	1.96
Imidacloprid			BQL	1.87	1.87	2.18	20%	0.351	1.05
Imidacloprid-Guanidine			BQL	0.408	0.408	0.408	9%	0.272	0.816
Omethoate			BQL	BQL	BQL	BQL	3%	0.357	1.07
Pyridaben			3.24	3.24	3.24	3.24	3%	0.676	2.03
Spinosad A (Spinosyn A)			BQL	BQL	BQL	BQL	3%	2.35	7.04
Sulfoxaflor			BQL	0.590	0.880	1.65	37%	0.149	0.446
Thiacloprid			0.392	0.392	0.392	0.392	3%	0.119	0.356
Thiamethoxam			BQL	3.69	3.69	7.08	6%	0.197	0.591
3-Keto-Carbofuran		Pesticides	BQL	BQL	BQL	BQL	97%	0.290	0.871
Diphenamid			BQL	0.373	0.489	1.22	43%	0.0792	0.237
Methiocarb-Sulfone			BQL	1.08	3.88	9.51	14%	0.212	0.635
2.3.4.6-Tetrachlorophenol			BQL	1.89	2.44	8.27	49%	0.603	1.81

Analyte Name	Classification	Sub class	Minimum (ng/L)	Median (ng/L)	Average (ng/L)	Maximum (ng/L)	% FoD	LOD (ng/L)	LOQ (ng/L)
Pentachlorophenol			BQL	0.278	0.459	1.74	63%	0.0489	0.147
Benzododecinium	Surfactants	Antiseptics & Disinfectants	1.99	11.9	23.6	260	97%	0.0520	0.156
Benzyldimethyltetradecylammonium			BQL	0.917	1.23	6.74	83%	0.0561	0.168

Table S.5. Determined analytes in post-flood **sediment** samples (N=25) collected in 2023. **Abbreviations:** BQL: Below Quantification Limit, FoD: Frequence of Detection

Analyte Name	Classification	Sub class	Minimum (µg/kg d.w.)	Median (µg/kg d.w.)	Average (µg/kg d.w.)	Maximum (µg/kg d.w.)	% FoD	LOD (µg/kg d.w.)	LOQ (µg/kg d.w.)
Caffeine	Coffee & Tobacco Related Compounds	Stimulants	4.83	7.18	9.86	18.7	19%	1.26	3.78
Nicotine			2.51	5.52	8.63	27.0	33%	0.759	2.28
Harman			0.761	1.80	1.76	2.67	33%	0.227	0.682
Cotinine			6.02	98.7	72.6	113	11%	1.82	5.47
Anabasine			BQL	BQL	BQL	BQL	4%	1.75	5.25
Acesulfame	Food Additives	Artificial Sweeteners	BQL	BQL	BQL	BQL	19%	0.177	0.530
Cyclamic acid			17.2	22.6	22.6	28.0	7%	1.68	5.04
Saccharin			5.03	5.03	5.03	5.03	4%	0.500	1.50
Sucralose			0.510	0.865	0.843	1.13	22%	0.129	0.388
5-MeO-DMT	Illicit Drugs	Psychedelic Drugs	1.06	1.28	3.29	7.51	16%	0.114	0.341
DEHP [Bis-(2-ethylhexyl) phthalate]	Industrial Chemicals & TPs	Plasticizers	36.2	77.1	86.0	182	81%	1.89	5.66
Di-n-octylphthalate (DOP)			3.23	50.8	55.5	197	85%	0.245	0.735
Diethyl phthalate (DEP)			10.3	75.9	71.1	186	81%	1.89	5.68
Triphenylphosphate			0.138	2.52	3.61	10.3	67%	0.0363	0.109
Benzyl butyl phthalate (BBP)			16.7	16.7	16.7	16.7	22%	0.502	1.51
Dimethylphthalate			0.138	0.190	0.287	0.778	26%	0.0426	0.128
Perfluorooctanoic acid (L-PFOA)		PFASs	BQL	BQL	BQL	BQL	11%	0.736	2.21
6:2 Fluorotelomer sulfonic acid (6:2			0.511	5.89	5.96	26.4	100%	0.122	0.366

Analyte Name	Classification	Sub class	Minimum (µg/kg d.w.)	Median (µg/kg d.w.)	Average (µg/kg d.w.)	Maximum (µg/kg d.w.)	% FoD	LOD (µg/kg d.w.)	LOQ (µg/kg d.w.)
FTS)									
Perfluorooctanesulfonic acid (PFOS)			2.03	13.0	17.4	104	100%	0.372	1.12
Perfluorononanoic acid (PFNA)			BQL	BQL	BQL	BQL	4%	0.823	2.47
Perfluorodecanoic acid (PFDA)			BQL	BQL	BQL	BQL	7%	0.211	0.634
Perfluorobutanoic acid (PFBA)			3.92	6.60	7.04	12.3	52%	1.29	3.88
Perfluoroundecanoic acid (PFUdA)			0.619	1.49	1.58	2.79	22%	0.196	0.588
Perfluoropentanoic acid (PFPeA)			1.53	2.58	2.61	3.44	26%	0.462	1.39
4,8-Dioxa-3H-perfluorononanoic acid (ADONA)			1.03	1.03	1.03	1.03	11%	0.189	0.567
2,3,3,3-Tetrafluoro-2-(heptafluoropropoxy)propanoic acid (HFPO-DA)			2.36	2.36	2.36	2.36	7%	0.605	1.81
Perfluoroheptanoic acid (PFHpA)			5.08	7.81	7.81	10.5	7%	0.353	1.06
Perfluorohexanoic acid (PFHxA)			BQL	BQL	BQL	BQL	4%	0.0938	0.281
Perfluorododecanoic acid (PFDoA)			1.08	1.49	2.16	6.32	67%	0.292	0.877
Perfluorooctanesulfonamide (PFOSA)			BQL	BQL	BQL	BQL	4%	0.372	1.12
Octocrylene	Personal Care Products & TPs	Sunscreen Components	2.17	3.56	3.68	5.91	74%	0.531	1.59
Galaxolidone		Synthetic Fragrances	4.05	7.64	9.35	21.2	37%	0.986	2.96
Lauramidopropylbetaine		Skin Cleansers	0.674	3.52	3.74	8.02	85%	0.159	0.478
Lauryl diethanolamide			1.08	4.07	5.68	16.5	56%	0.307	0.920
Triclosan			1.39	1.39	1.39	1.39	4%	0.385	1.15

Analyte Name	Classification	Sub class	Minimum (µg/kg d.w.)	Median (µg/kg d.w.)	Average (µg/kg d.w.)	Maximum (µg/kg d.w.)	% FoD	LOD (µg/kg d.w.)	LOQ (µg/kg d.w.)
Triclocarban			0.399	0.453	0.647	1.09	11%	0.102	0.307
Metoprolol	Pharmaceuticals & TPs	Antihypertensives	2.69	2.69	2.69	2.69	41%	0.289	0.868
Minoxidil			32.5	32.5	32.5	32.5	7%	1.38	4.13
Apophedrin (Phenylethanolamine)			3.89	11.2	12.6	22.5	78%	1.08	3.25
Propafenone		Antiarrhythmics	BQL	BQL	BQL	BQL	7%	0.267	0.801
Salicylic acid		Analgesics & Anaesthetics	2.27	4.92	9.27	34.2	89%	0.736	2.21
Ketamine			0.538	0.538	0.538	0.538	7%	0.115	0.345
Oxyclozanide		Anthelmintics	0.482	0.782	1.43	3.67	33%	0.146	0.439
Fenbendazole			89.5	89.5	89.5	89.5	4%	1.92	5.76
Flubendazole			BQL	BQL	BQL	BQL	4%	0.230	0.691
Sulfachloropyridazine		Antibiotics	0.518	1.28	1.95	6.30	26%	0.129	0.387
Sulfaethoxypyridazine			5.99	25.8	25.8	45.6	7%	0.240	0.730
Orbifloxacin			9.06	9.06	9.06	9.06	4%	2.52	7.55
Cinoxacin			2.32	2.32	2.32	2.32	7%	0.357	1.07
Cimetidine		Antiulcers	36.6	36.6	36.6	36.6	4%	2.60	7.80
Ketotifen		Antihistamine Agents	BQL	BQL	BQL	BQL	4%	0.262	0.786
Cinnarizine			BQL	BQL	BQL	BQL	4%	0.808	2.42
Bisoprolol		Beta Blockers	BQL	BQL	BQL	BQL	7%	0.195	0.584
Bunitrolol			11.7	29.3	66.1	214	19%	3.00	9.00
Ketoprofen		NSAIDs	0.820	1.03	1.05	1.29	19%	0.182	0.547
Flunixin			BQL	BQL	BQL	BQL	4%	0.247	0.742
Ibuprofen			BQL	BQL	BQL	BQL	7%	1.85	5.55

Analyte Name	Classification	Sub class	Minimum (µg/kg d.w.)	Median (µg/kg d.w.)	Average (µg/kg d.w.)	Maximum (µg/kg d.w.)	% FoD	LOD (µg/kg d.w.)	LOQ (µg/kg d.w.)
Phenylbutazone		Psychoactive Substances	3.78	4.08	4.08	4.38	11%	0.948	2.84
Tolfenamic acid			BQL	BQL	BQL	BQL	4%	0.109	0.327
Amisulpiride			3.69	3.69	3.69	3.69	7%	0.737	2.21
Hordenine		Stimulants	9.34	96.3	156	424	15%	0.338	1.02
Florfenicol		Veterinary Drugs	0.106	0.109	0.128	0.169	52%	0.0347	0.104
Olaquindox			BQL	BQL	BQL	BQL	4%	0.956	2.87
N.N-Diethyl-m-toluamide. DEET	Plant Protection Products & TPs	Insecticides	0.864	0.864	0.864	0.864	52%	0.278	0.833
Clothianidin			0.157	0.157	0.157	0.157	7%	0.0481	0.144
Dinotefuran			BQL	BQL	BQL	BQL	4%	0.0280	0.0840
Ethiofencarb-sulfone			BQL	BQL	BQL	BQL	4%	0.300	0.899
Fenthion-sulfoxide			BQL	BQL	BQL	BQL	4%	0.0501	0.150
Carbofuran 3-keto-		Pesticides	0.290	0.332	0.347	0.419	37%	0.0952	0.286
Pentachlorophenol. PCP			0.406	1.01	0.988	1.44	26%	0.104	0.312
Carbamate Ethyl-N-(3-hydroxyphenyl)-			0.992	1.80	1.98	3.21	30%	0.255	0.766
Fipronilsulfone			0.338	0.338	0.338	0.338	4%	0.0870	0.260
Azoxystrobin		Fungicides	0.0704	0.113	0.152	0.307	30%	0.0163	0.0488
Boscalid			0.226	0.497	0.587	1.21	22%	0.0259	0.0778
BTS 44596 Fragm 195 (Prochloraz metabolite)			2.08	2.16	2.83	4.25	11%	0.154	0.461
Carbendazim			0.134	0.180	0.451	1.04	11%	0.0344	0.103
Chlorothalonil-4-hydroxy			0.201	0.809	1.16	3.70	44%	0.0335	0.101

Analyte Name	Classification	Sub class	Minimum (µg/kg d.w.)	Median (µg/kg d.w.)	Average (µg/kg d.w.)	Maximum (µg/kg d.w.)	% FoD	LOD (µg/kg d.w.)	LOQ (µg/kg d.w.)
Dichlorophen		Herbicides	0.236	0.772	0.619	0.848	11%	0.0179	0.0537
Fludioxonil			0.233	0.239	0.239	0.244	7%	0.0578	0.173
Fluometuron			0.143	0.171	0.183	0.235	15%	0.0189	0.0567
Prometryn (Caparol)			0.171	0.171	0.171	0.171	11%	0.0563	0.169
Ioxynil			0.0400	0.308	0.377	1.02	63%	0.0114	0.0341
Phenmedipham metabolite MHPC			BQL	BQL	BQL	BQL	19%	0.0837	0.251
Metolachlor			0.453	0.473	0.473	0.492	19%	0.0797	0.239
Difenzoquat			10.4	15.5	35.6	98.8	33%	0.311	0.932
Carbanilide		Growth Regulators	1.02	2.00	1.73	2.16	11%	0.110	0.330
DDAC-C10	Surfactants	Antiseptics & Dissinfectants	0.149	0.405	0.399	0.679	52%	0.0165	0.0494
Benzyldimethylhexadecylammonium			0.194	0.323	0.320	0.578	41%	0.0402	0.121
N,N-Dimethyltetradecylamine-N-oxide			0.0469	0.376	0.574	1.56	56%	0.0120	0.0361
Benzyldimethyltetradecylammonium			0.260	0.657	0.768	1.93	52%	0.0713	0.214
N-Methyldodecylamine			0.277	0.453	0.453	0.629	7%	0.0185	0.0554
N,N-Dimethyldodecylamine N-oxide			0.336	0.431	0.511	0.847	19%	0.101	0.302

To compare the environmental status of the studied gulf with a prior condition, unpublished data from samples collected in 2020 were considered herein. Compounds determined during the 2020 sampling campaign are presented in the following tables (Table S6 and Table S7):

Table S.6. Determined analytes in **seawater** samples (N=2) prior to the flood events (2020). **Abbreviations:** BQL: Below Quantification Limit

Analyte Name	Classification	Sub class	Volos Port (St. 12) (ng/L)	Volos (St. 13) (ng/L)	LOD (ng/L)	LOQ (ng/L)	%FoD
Caffeine	Coffee & Tobacco related compounds	Stimulants	BQL	8.26	0.230	0.689	100%
Nicotine			BQL	171	0.0413	0.124	100%
Theobromine			BQL	1.94	0.202	0.605	100%
Cotinine			BQL	4.16	0.186	0.559	100%
Cotinine-Hydroxy			BQL	0.201	0.248	0.743	100%
2-OH-Benzothiazole	Industrial Chemicals & TPs	Corrosion Inhibitors	BQL	5.89	0.685	2.05	100%
1-H-Benzotriazole (BTR)			BQL	1.42	0.0917	0.275	100%
2-Amino-Benzothiazole-			<LOD	8.68	0.223	0.670	33%
Mixture of 4- and 5- Me-Benzotriazole			BQL	BQL	0.685	2.05	100%
Perfluoroheptanoic acid (PFHpA)		PFAS	0.134	1.66	0.485	1.46	100%
Perfluorohexanoic acid (PFHxA)			BQL	1.50	0.233	0.698	100%
Bisphenol A		Plastic Production	<LOD	3.58	0.302	0.906	33%
Paracetamol	Pharmaceuticals & TPs	Analgesics & Anaesthetics	<LOD	15.4	2.15	6.45	33%
Sulfadiazine		Antibiotics	BQL	2.88	0.201	0.604	100%
Trimethoprim			7.90	<LOD	0.118	0.354	33%
Lamotrigine		Antiepileptics	0.556	0.694	0.122	0.365	67%
Atenolol		Antihypertensives	<LOD	11.6	0.294	0.882	33%
Venlafaxine-O-Desmethyl		Psychoactive Substances	BQL	BQL	0.191	0.572	100%
Atrazine	Plant Protection Products & TPs	Herbicides	0.231	<LOD	0.108	0.323	33%
Atrazine-2-hydroxy			0.368	0.937	0.0451	0.135	67%
Dinoterb			BQL	1.15	0.0900	0.270	100%

Analyte Name	Classification	Sub class	Volos Port (St. 12) (ng/L)	Volos (St. 13) (ng/L)	LOD (ng/L)	LOQ (ng/L)	%FoD
Fluometuron			<LOD	<LOD	0.0410	0.124	0%
Propazine-2-hydroxy			0.349	0.460	0.162	0.485	67%
Boscalid		Fungicides	1.54	13.9	0.0917	0.275	67%
Dinitrophenol-2-4- (DNP)		Pesticides	3.67	7.20	0.603	1.81	100%
Tributylamine		Insecticides	BQL	15.3	0.104	0.312	100%

Table S.7. Determined analytes in **sediment** samples (N=2) prior to the flood events (2020). **Abbreviations:** BQL: Below Quantification Limit

Analyte Name	Classification	Sub class	Volos (St. 13)	Peneus river delta out (St. 3)	LOD (μg/kg d.w.)	LOQ (μg/kg d.w.)	%FoD
			(μg/kg d.w.)	(μg/kg d.w.)			
Anabasine	Coffee & Tobacco related compounds	Stimulants	35.6	47.6	1.75	5.83	100%
Cotinine			10.3	12.6	1.82	6.07	100%
Nicotine			20.3	29.1	0.759	2.53	100%
Theobromine			298	80.1	3.95	13.2	100%
Dibromo-4-hydroxy-benzoic acid 3-5-	Industrial Chemicals & TPs	Paraben Precursors	32.4	9.19	0.174	0.580	100%
Perfluorooctanesulfonic acid (PFOS)		PFASs	3.10	6.91	0.372	1.24	100%
Perfluorooctanoic acid (PFOA)			BQL	<LOD	0.736	2.45	50%
Perfluoroheptanoic acid (PFHpA)			<LOD	4.02	0.353	1.18	50%
Benzyl butyl phthalate (BBP)		Plasticizers	22.3	14.9	0.502	1.67	100%
DEHP [Bis-(2-ethylhexyl) phthalate]			33.0	43.3	1.89	6.30	100%
Di-n-octylphthalate (DOP)			14.6	18.4	0.245	0.817	100%
Triphenylphosphate			15.5	BQL	0.363	1.21	100%
Ketamine	Pharmaceuticals & TPs	Analgesics & Anaesthetics	7.70	7.16	0.115	0.383	100%
Paracetamol			<LOD	34.5	2.47	8.22	50%
Salicylic acid			12.3	14.0	0.736	2.45	100%
Fenbendazole		Anthelmintics	11.0	9.59	1.92	6.40	100%
Embutramide		Antibiotics	38.5	41.3	2.14	7.13	100%
Venlafaxine		Antidepressants	4.25	BQL	0.579	1.93	100%
Levetiracetam		Antiepileptics	BQL	BQL	2.38	7.93	100%
Losartan		Antihypertensives	BQL	BQL	5.35	17.8	100%

Analyte Name	Classification	Sub class	Volos (St. 13) (μg/kg d.w.)	Peneus river delta out (St. 3) (μg/kg d.w.)	LOD (μg/kg d.w.)	LOQ (μg/kg d.w.)	%FoD
Cimetidine		Antiulcers	10.6	BQL	2.60	8.67	100%
Ondansetron		Chemotherapy Agents	0.544	BQL	0.163	0.544	100%
Hydrochlorothiazide		Diuretics	<LOD	0.752	0.0418	0.139	50%
Tolfenamic acid		NSAIDs	BQL	9.13	0.109	0.363	100%
Triclopyr	Plant Protection Products & TPs	Herbicides	BQL	5.73	0.491	1.64	100%
N.N-Diethyl-m-toluamide. DEET		Insecticides	BQL	BQL	0.278	0.927	100%
Benzododecinium	Surfactants	Antiseptics & Disinfectants	5.05	BQL	0.389	1.30	100%

References

[1] N.A. Alygizakis, P. Gago-Ferrero, V.L. Borova, A. Pavlidou, I. Hatzianestis, N.S. Thomaidis, Occurrence and spatial distribution of 158 pharmaceuticals, drugs of abuse and related metabolites in offshore seawater, Science of the Total Environment, vol. 541, 2016, pp. 1097–1105.

[2] V. Nikolopoulou, N.A. Alygizakis, M.C. Nika, M. Oswaldova, P. Oswald, M. Kostakis, A. Koupa, N.S. Thomaidis, J. Slobodnik, Screening of legacy and emerging substances in surface water, sediment, biota and groundwater samples collected in the Siverskyi Donets River Basin employing wide-scope target and suspect screening, Science of the Total Environment, vol. 805, 2022,.

[3] S.K. Drakopoulou, A.S. Kritikou, C. Baessmann, N.S. Thomaidis, Untargeted 4D-metabolomics using Trapped Ion Mobility combined with LC-HRMS in extra virgin olive oil adulteration study with lower-quality olive oils, Food Chemistry, vol. 434, 2024, pp. 137410.

[4] S.K. Drakopoulou, D.E. Damalas, C. Baessmann, N.S. Thomaidis, Trapped Ion Mobility Incorporated in LC-HRMS Workflows as an Integral Analytical Platform of High Sensitivity: Targeted and Untargeted 4D-Metabolomics in Extra Virgin Olive Oil, Journal of Agricultural and Food Chemistry, vol. 69, 2021, pp. 15728–15737.

[5] N. Thomaidis, C. Baessmann, D. Damalas, K. Diamanti, B. Galvin, M. Georgakaki, G. Gkotsis, A. Konomi, R. Lougkovois, M.-C. Nika, E. Panagopoulou, I. Routsai, B. Schneider, K. Wendt, National and Kapodistrian University of Athens LC-TIMS-HRMS Target List, https://doi.org/10.5281/zenodo.13862246
